# Computational analysis of androgen receptor (AR) variants to decipher the relationship between protein stability and related-diseases

**DOI:** 10.1038/s41598-020-68731-7

**Published:** 2020-07-21

**Authors:** Fangfang Chen, Xiaoqing Chen, Fan Jiang, Feng Leng, Wei Liu, Yaoting Gui, Jing Yu

**Affiliations:** 1 Guangdong and Shenzhen Key Laboratory of Male Reproductive Medicine and Genetics, Peking University Shenzhen Hospital, Shenzhen PKU-HKUST Medical Center, Shenzhen, 518036 China; 2 Department of Orthopaedics, Quanzhou First Hospital Affiliated to Fujian Medical University, Quanzhou, 350005 China; 3 NanoAI Biotech Co., Ltd., Huahan Technology Industrial Park, Pingshan District, Shenzhen, 518109 China; 4 National Cancer Institute, National Institute of Health, Bethesda, MD 20892 USA; 5 Shenzhen Key Laboratory for Neuronal Structural Biology, Biomedical Research Institute, Shenzhen PKU-HKUST Medical Center, Shenzhen, 518036 China; 6 Department of Laboratory Medicine, Peking University Shenzhen Hospital, Shenzhen, 518036 China

**Keywords:** Reproductive disorders, Sexual dysfunction

## Abstract

Although more than 1,000 androgen receptor (AR) mutations have been identified and these mutants are pathologically important, few theoretical studies have investigated the role of AR protein folding stability in disease and its relationship with the phenotype of the patients. Here, we extracted AR variant data from four databases: ARDB, HGMD, Cosmic, and 1,000 genome. 905 androgen insensitivity syndrome (AIS)-associated loss-of-function mutants and 168 prostate cancer-associated gain-of-function mutants in AR were found. We analyzed the effect of single-residue variation on the folding stability of AR by FoldX and guanidine hydrochloride denaturation experiment, and found that genetic disease-associated mutations tend to have a significantly greater effect on protein stability than gene polymorphisms. Moreover, AR mutants in complete androgen insensitivity syndrome (CAIS) tend to have a greater effect on protein stability than in partial androgen insensitive syndrome (PAIS). This study, by linking disease phenotypes to changes in AR stability, demonstrates the importance of protein stability in the pathogenesis of hereditary disease.

## Introduction

Since most proteins need to be folded to function, protein stability is one of the most basic properties of a protein. The protein stability discussed herein primarily refers to the thermodynamic stability of a protein, which determines whether the protein is in a naturally folded configuration or a denatured (unfolded or extended) state. Protein stability is a fundamental property that affects protein configuration, activity and regulation. It plays an essential role in evolution, a variety of diseases and industrial applications^1–4^. The most common cause of monogenic diseases is single-nucleotide variation (SNV) leading to amino acid substitutions. These missense variants can have a strong effect on the stability of a protein, leading to detrimental changes to protein function. Loss of protein stability is a major contributor to this single-gene disease^1^. More and more attention has been paid in the past few decades to understand the biological principles of protein stability^5,6^. Accurately predicting protein stability through theoretical and experimental methods is crucial for academic research and industrial applications.

Androgens have a wide range of physiological effects on male reproductive and non-reproductive systems at different stages of development^7–9^. During the fetal period, androgens are primarily responsible for sex differentiation by masculinizing the Wolff tube and external genitalia^9^. During puberty, androgens regulate the growth and function of the male reproductive system^9^. In adults, androgens play key role in regulating behavior, spermatogenesis and bone metabolism^9^. Androgens mediate their actions primarily via the androgen receptor (AR), a ligand-dependent nuclear transcription factor expressed in primary/secondary sex organs^8,9^. AR is also expressed in non-genital organs such as the skeletal muscle, skin, adrenal gland, kidney and nervous system^8,9^.

AR is an extensively studied steroid receptors. AR mutations have been identified in various diseases, including hereditary diseases such as androgen insensitivity syndrome (AIS)^10,11^, spinal and bulbar muscular atrophy (SBMA)^12,13^ and benign prostatic hyperplasia^14,15^. AR also plays an important role in the development and metastasis of several hormone-related cancers, including prostate cancer^12^, breast cancer^16,17^, liver cancer^18–20^.

The AR contains three major functional domains: (1) the N-terminal domain (NTD) comprises an activation function 1 (AF-1) region, (2) DNA binding domain (DBD) and (3) the C-terminal ligand binding domain (LBD) comprise an AF-2 region^8,21^. The primary mechanism of action for AR is to directly regulate gene transcription. Androgen binds to the AR, leading to conformational change of AR, dissociation of heat shock proteins, driving the interaction between the N- and C-terminus of AR, and importin-α binds AR to transport AR into the nucleus^22^. In the nucleus, the AR dimerizes and binds to androgen response elements (ARES) in the promoter region of the target genes^23^. AR interact with additional proteins in the nucleus, causing the transcription of specific target genes to be up- or down-regulated. Notable target genes for AR are insulin-like growth factor I receptor (IGF-1R)^24^, prostate-specific antigen (PSA)^25,26^, and transmembrane protease serine 2 (TMPRSS2)^27–29^.

Although more than 1,000 AR mutations have been identified and these mutants are pathologically important^12^, few theoretical studies have investigated the impact of mutations on AR protein folding stability in disease and its relationship with the phenotype of the patients. Several algorithms have been developed to predict the effect of mutations on protein stability^30–35^. Notable algorithms include FoldX^36^, Dmutant^37^, I-Mutant2.0^38^, CUPSAT^39^, Eris^40^ and STRUM^41^. Compared with other methods, FoldX performs well and is the most commonly used protein stability prediction algorithm^42^. Folding free energy reflects the overall protein stability, and changes in protein stability due to naturally occurring missense mutations often cause disease^42^. This work calculates the folding free energy of AR variants by FoldX and measures the guanidine hydrochloride (GdmHCl) denaturation curves of different mutants, trying to establish correlation between protein stability and patient phenotype. By correlating the patient's phenotype with changes in AR stability, this study may prove to be diagnostic and/or predictive tools for assessing the effects of mutations on disease outcome.

## Results and discussion

### Computational pipeline for disease-associated androgen receptor (AR) variants

**Figure 1:**
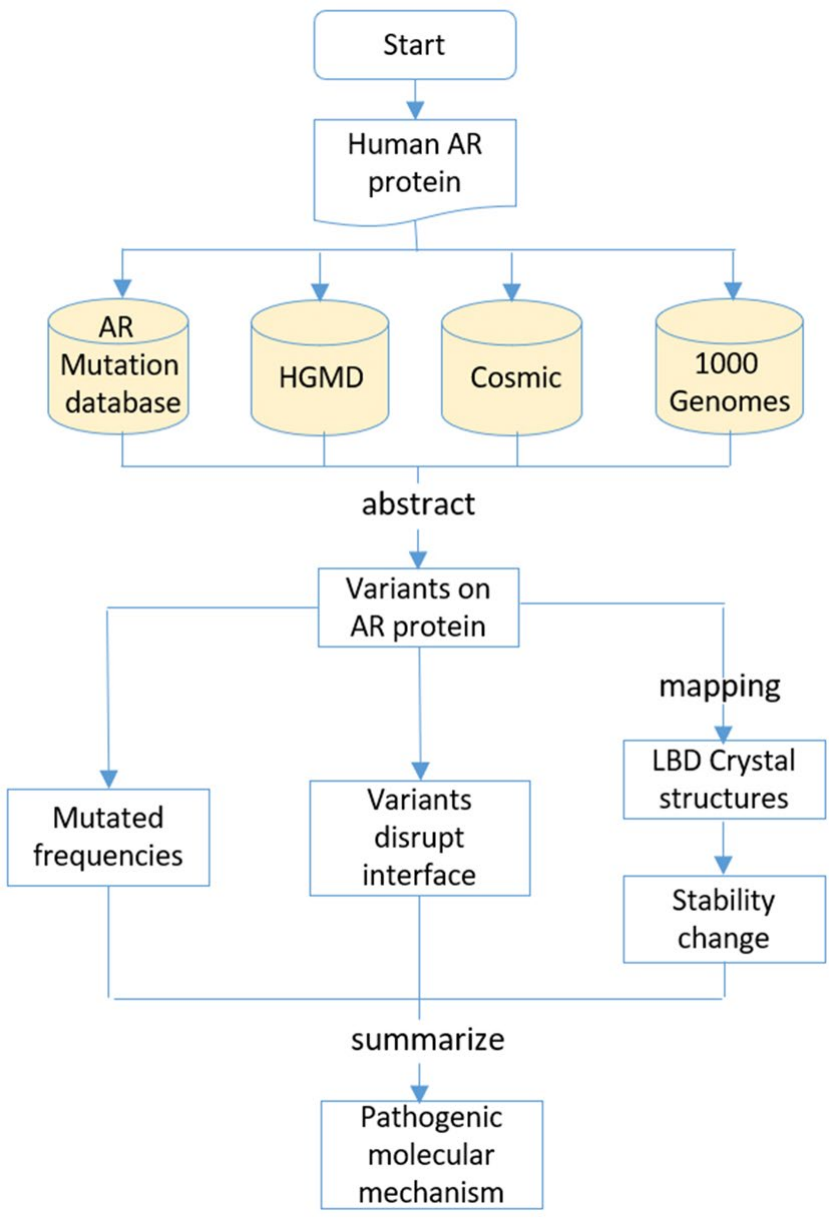
Flowchart of the computational analysis pipeline for disease-associated androgen receptor (AR) variants.

In order to study the pathogenic pattern of AR mutants in related diseases, our computational analysis consist of the following steps as in Fig. 1: (1) extracted AR variations from the following four databases: ARDB (The androgen receptor gene mutations database: 2012 update; https://androgendb.mcgill.ca), HGMD (Human Gene Mutation Database, 2015), Cosmic (2018.10.01), 1,000 genome; (2) analyzed mutation frequency, protein stability and relative surface accessibility (RSA) of these variants; (3) summarized the pathogenic mechanism of AR mutants in related diseases.

### AR structure

**Figure 2:**
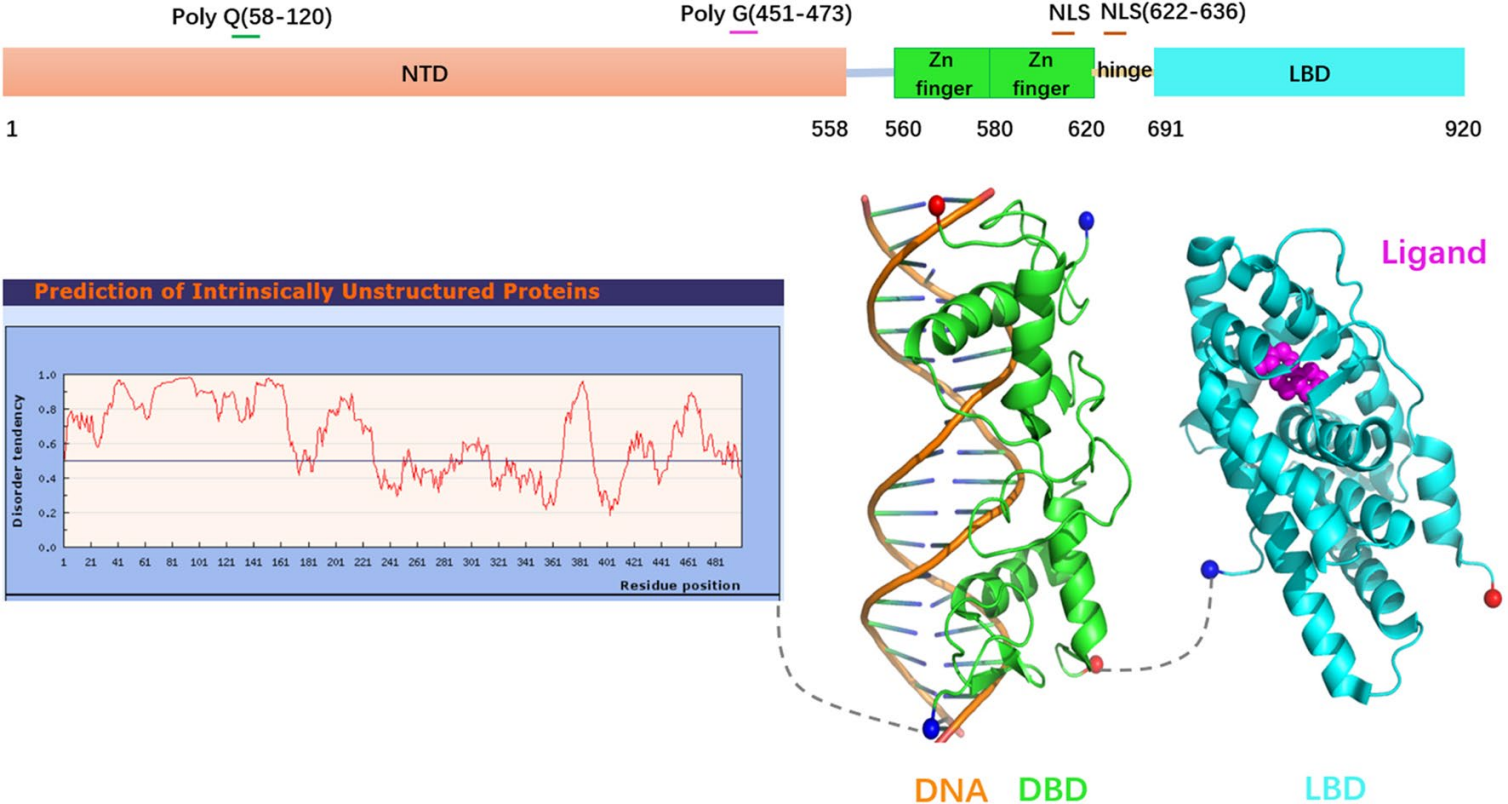
AR domains and structure. Blue and red spheres represent N-terminal and C-terminal, respectively.

The NTD domain accounts for more than half of the entire AR protein as in Fig. 2 (amino acids 1–558). AR NTD contains polyglutamine (ploy-Q) and polyglycine (poly-G) repeats, and the length of these two repeats is highly variable in the human population^43,44^. The latest human AR reference gene sequence (NM_000044.2) encodes a protein of 920 amino acids in length (instead of the previous 919). Because the reference length of ploy-Q is replaced by 23 instead of the original 21, and the reference length of poly-G is changed from 24 to 23. The length of the ploy-Q repeat is inversely proportional to the AR transcriptional activity, and the longer the polyglutamine repeat, the smaller the AR transcriptional activity^45^. The AR NTD is an intrinsically disordered proteins (IDP) that lacks a stable structure in aqueous solution (Fig. 2)^46,47^. AR NTD undergoes conformational changes when interacting with DNA and/or target proteins and in the presence of structurally stable solutes^46,47^. The plasticity of the AR NTD structure allows it to interact with many structurally distinct proteins (e.g., P160 family coactivator, transcription factor IIF) and intramolecularly interact with C terminal LBD domain (N/C interaction of AR)^48–51^.

AR DBD (amino acids 560–620) consists of two zinc-coordinated modules, and it is highly conserved among steroid hormone receptors (Fig. 2). DBD selectively binds to the androgen response elements (ARES) on the promoter, activating specific AR target genes, such as TMPRSS2, PSA and IGF-1R^52^. AR contains two NLS sequences-one in the DBD domain, the other one in the hinge region between DBD and LBD. NLS consists of two basic amino acid clusters separated by ten residues (617-RKCYEAGMTLGARKLKK-634). The binding of androgens to AR induces the exposure of NLS and result in nuclear import of AR by binding to the importin-α^53^.

The crystal structure of AR LBD (residues 691–920) was first well characterized in 2000^54^. Subsequently, many related complex structures were deposited to the RCSB PDB (The Research Collaboratory for Structural Bioinformatics Protein Data Bank). AR LBD consists of 11 α-helices and 4 short β-strands, forming a three-layer anti-parallel α-helical sandwich fold, which is characteristic of AR LBD (Fig. 2)^54^.

### AR mutations analysis

#### Relationship between AR mutations and diseases

1,110 mutations were found in the AR gene, of which 905 were possible loss-of-function alterations and caused androgen insensitivity syndrome (AIS) or related with AIS. Different AR mutations impair androgen-dependent male sexual differentiation to varying degrees^12^. Severe androgen insensitivity (AI) produces an external female phenotype. Partial AI produce a range of external genital phenotypes, from near-normal females to normal or near-normal males. It has been suggested that the clinical severity of AI be divided into three levels: complete, partial (when there is significant external genital ambiguity), and mild (for the least severe form). In addition, there are 4 AR loss-of-function mutations associated with premature ovarian failure (POF)^12^.

There are also 168 AR mutations that are possible gain-of-function alterations found in prostate cancer tissues. Aside from skin cancer, prostate cancer is the most common cancer among men and is the second leading cause of cancer-related death in the United States^55^. In prostate cancer, AR mutation may reduce the specificity and selectivity of its binding ligands, thereby being activated by a wider range of ligands such as adrenal androgens, estrogens, progesterone and antiandrogens. These gain-of-function AR mutations in prostate cancer tissue may be responsible for the failure of prior anti-androgen therapy. In addition, in spinal cord and bulbar muscular atrophy (SBMA, also known as Kennedy's disease), poly-Q amplified AR protein is toxic to motor neurons to some extent by gain-of-function^56^.

#### AR mutations statistics

**Figure 3:**
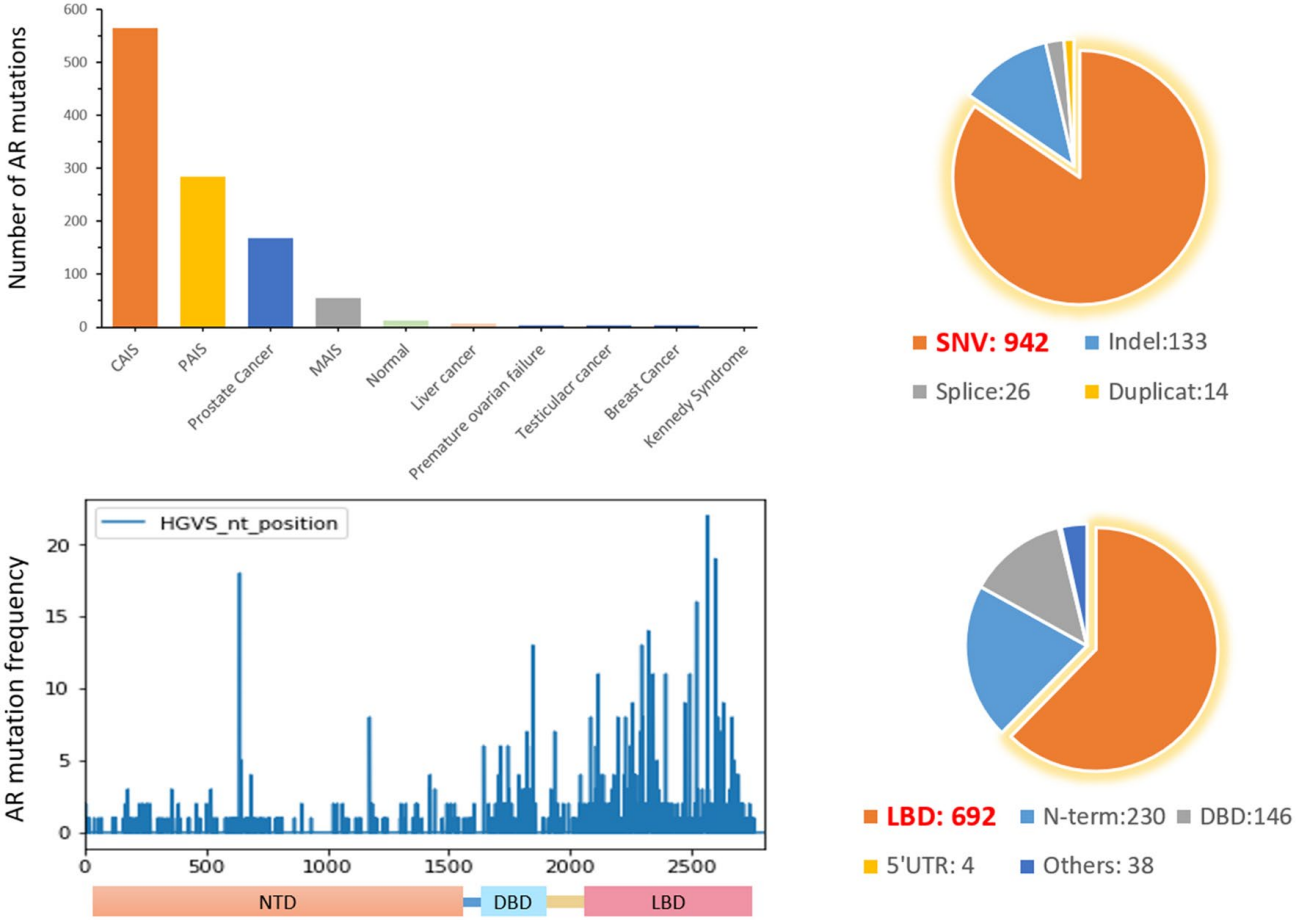
Analysis of AR mutations from three datasets.

The AR NTD is highly conserved with relatively few deleterious mutations, and only 8% of the residues in the domain are found to have mutations, including 230 variants (Fig. 3). Most mutations are nonsense mutations or frameshift mutations result in premature termination of the translation. A significantly higher percentage (27%) of residue mutations were found in the DBD domain, including 146 variants (Fig. 3). Mutations in DBD domain are primarily SNVs, result in defects in the DNA binding/dimerization activity of the protein and impaired or absent transcription activity of AR. In the hinge region of the AR, only 8% of the residues are found to have mutations. Some variations in this region have no significant adverse effects on AR function. In LBD domain, 56% of the residues contained mutations, including 692 variants (Fig. 3).

### The effect of disease-related AR SNV on protein stability

Nonsense or frameshift variants cause large changes to the encoded protein and are therefore usually functionally damaging. However, missense variants (single-residue variants, SRV), in which one amino acid is replaced by another amino acid, account for more than 40% of the unique variants observed in the Exome Aggregation Consortium database^57^, and their phenotypic consequences are often hard to predict. It has been found in cell experiments that many SRV have only a small effect on protein function. Analysis of high-throughput data across multiple proteins has shown that about two-thirds of SRV have only a small effect on protein function^58^. However, some SRV are severely harmful and cause complete loss of function. In a clinical setting, it would be useful to have reliable methods and sufficient data for interpretation of SRV and an accurate classification of whether they are pathogenic or benign.

**Figure 4:**
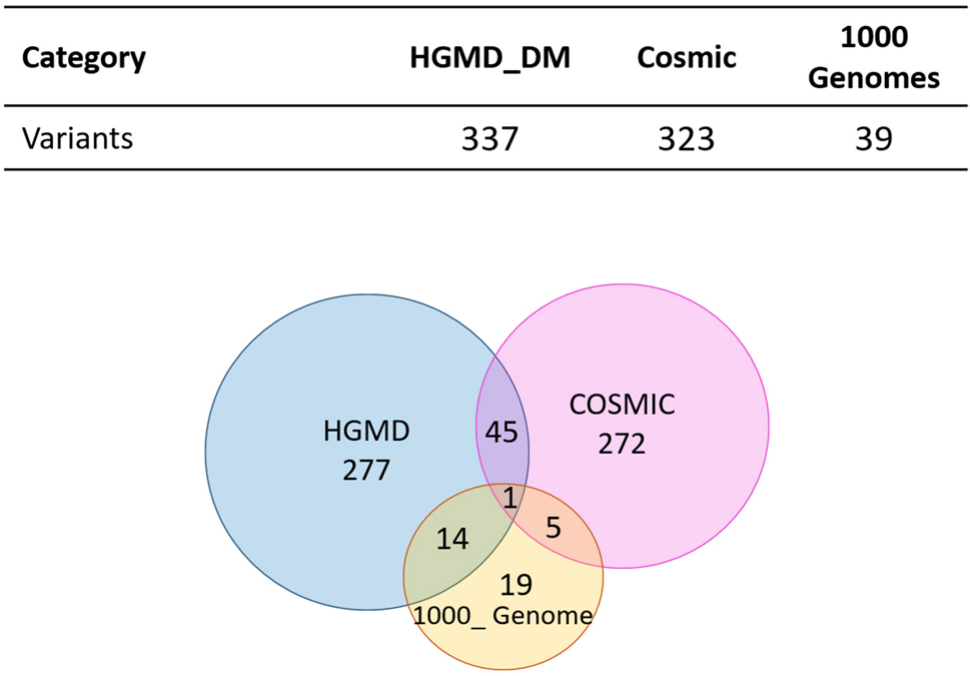
Venn diagram of the SRV variants in HGMD, COSMIC and 1,000 Genome datasets.

From the HGMD dataset, we downloaded a collection of 337 single residue mutations in the AR that are known to be associated with human inherited disease. All mutations in this set were found in patients with AIS and were associated with loss of AR function. From the Cosmic dataset, we downloaded 323 single residue mutations in the AR that are known to be associated with human cancers, the vast majority of which were associated with prostate cancer. From the 1,000 genome dataset, we downloaded 39 single residue variations in the AR that are not significantly associated with human diseases. The Venn diagram below shows the relations between these three AR mutation sets (Fig. 4).

#### The effect of disease-related AR mutants on protein folding stability

**Figure 5:**
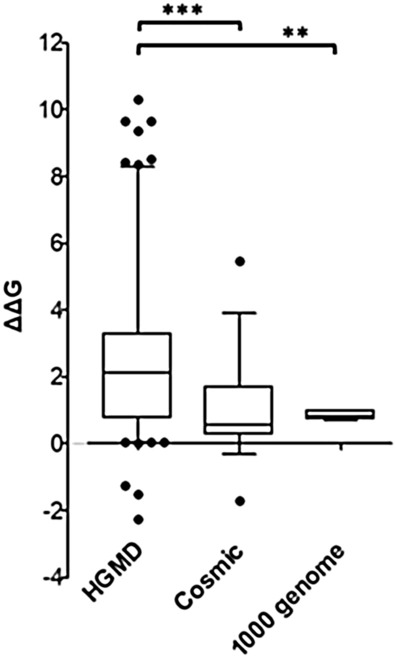
The predicted folding free energy change of AR LBD single residue variations calculated by FoldX. The statistical difference between HGMD, Cosmic and 1,000 genome groups was measured. The significance of statistical difference was calculated by paired two-side Student’s t-test (**p < 0.01, ***p < 0.001).

FoldX, the most commonly used protein stability prediction algorithm, can estimate the effect of SRV on protein stability based on the three-dimensional structure of the protein. So far, only the DBD and LBD domain in AR has crystal structure available, so we selected SRV of the DBD and LBD domain for analysis. We applied FoldX to analyze single residue variations in the AR DBD and LBD domain to estimate changes in protein folding free energy caused by these variations. We found that the predicted folding free energy change ΔΔG of human inherited disease-associated AR mutations (HGMD) was significantly higher (Fig. 5). The ΔΔG caused by the AR polymorphism (1,000 genome) that are not significantly related to the diseases are mainly around 1 kcal/mol. The average ΔΔG of tumor-associated AR somatic mutants is slightly lower than that caused by polymorphisms. However, mutations associated with human inherited diseases tend to have a significantly greater impact on protein stability than polymorphic or tumor-associated somatic mutants, with an average ΔΔG > 2 kcal/mol (Fig. 5).

Structurally unstable or misfolded proteins may form toxic aggregates or inclusions. Organisms control protein quality by refolding or degrading of these unstable or misfolded proteins^59^. Most intracellular protein degradation occurs via the ubiquitin–proteasome system (UPS) or autophagy–lysosomal pathway (ALP). In a folded protein, degradation signals are usually buried in the protein. When a protein is partially or fully unfolded, one or more degrons of the protein may be exposed. E3 ubiquitin ligase scans cells for such degradation signals, binds substrates, and promotes substrate ubiquitination and degradation by the 26S proteasome. A recent study on Lynch syndrome-associated MSH2 mutations found that, as little as 3 kcal/mol was sufficient to trigger protein degradation^60^. The ALP is usually responsible for the degradation of highly misfolded and insoluble protein aggregates.

#### Correlation between protein folding stability of AR mutation and AIS patient phenotype

**Figure 6:**
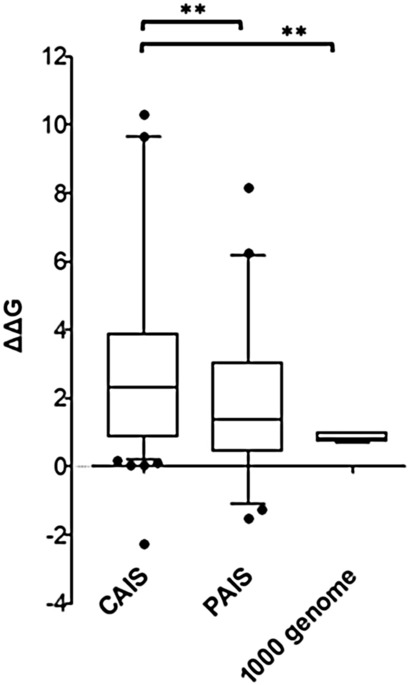
Correlation between protein folding stability of AR mutation and AIS patient phenotype. The statistical difference between CAIS, PAIS and 1,000 genome groups was measured. The significance of statistical difference was calculated by paired two-side Student’s t-test (**p < 0.01).

We further analyzed the correlation between the clinical severity of the AIS patient and the folding stability of the related AR mutation. AR mutants that cause severe androgen insensitivity (CAIS) tend to have a significantly greater impact on protein stability compared to partial AI (PAIS). PAIS AR mutants compared to polymorphisms tend to have a slight greater impact on protein stability (Fig. 6).

#### Expression and purification of AR-LBD WT and mutants

**Figure 7:**
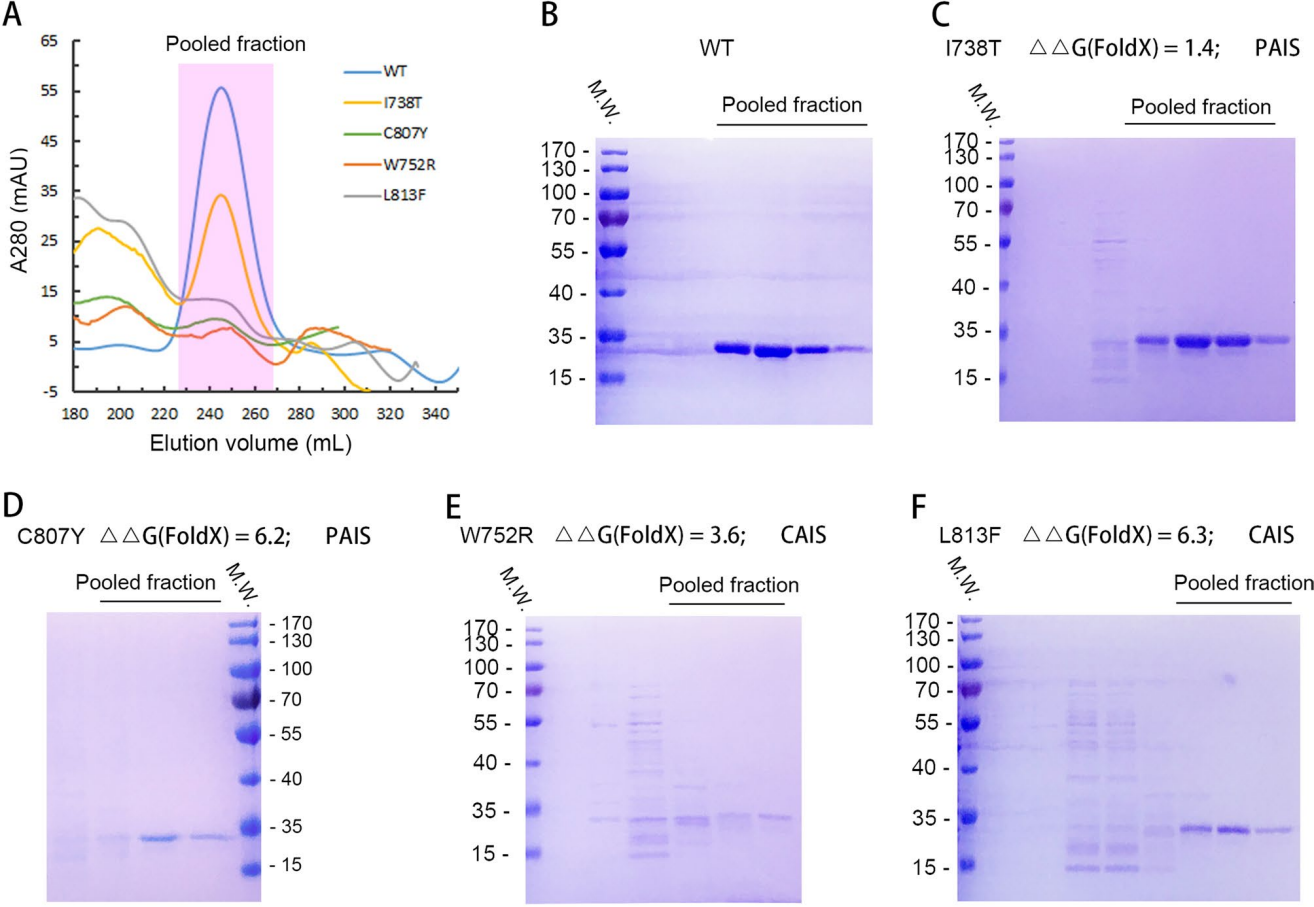
The expression and purification of his tag AR-LBD WT and I738T, C807Y, W752R, L813 mutant proteins. (**A**) Gel filtration profiles of AR-LBD WT and mutant proteins. AR-LBD WT and mutant protein was eluted at a peak of 250 mL in Superdex-75 column. (**B**–**F**) Coomassie blue stained SDS gel of the pooled fractions of AR-LBD WT and mutant proteins.

Besides the wide-type (WT) AR, we randomly select two AR mutants that cause CAIS (W752R, L813F) and two mutants that cause PAIS (I738T, C807Y). The above proteins with N-terminal his-tag were expressed in *Escherichia coli* and purified by Ni–NTA affinity column and following Superdex-75 gel filtration column (Fig. 7). Compared with other mutants, we found that the I738T mutant had small effect on its solubility (Fig. 7), in agreement with FoldX prediction that the I738T has a mild effect on protein folding stability (ΔΔG = 1.4 kcal/mol). FoldX predicts that ΔΔG of C807Y, W752R and L813 mutants are 6.2, 3.6 and 6.4 kcal/mol, respectively. Our experiments show that the solubilities of these three mutants are much lower than the WT (Fig. 7). We proposed that the poor solubilities of these three mutants may due to their decreased folding stabilities, therefore leading to high tendency to precipitate as inclusion bodies (data not shown). As will be shown later by GdmHCl-induced denaturation experiments, these three mutations indeed have severely problems in terms of folding stability.

#### GdmHCl-induced chemical denaturation

**Figure 8:**
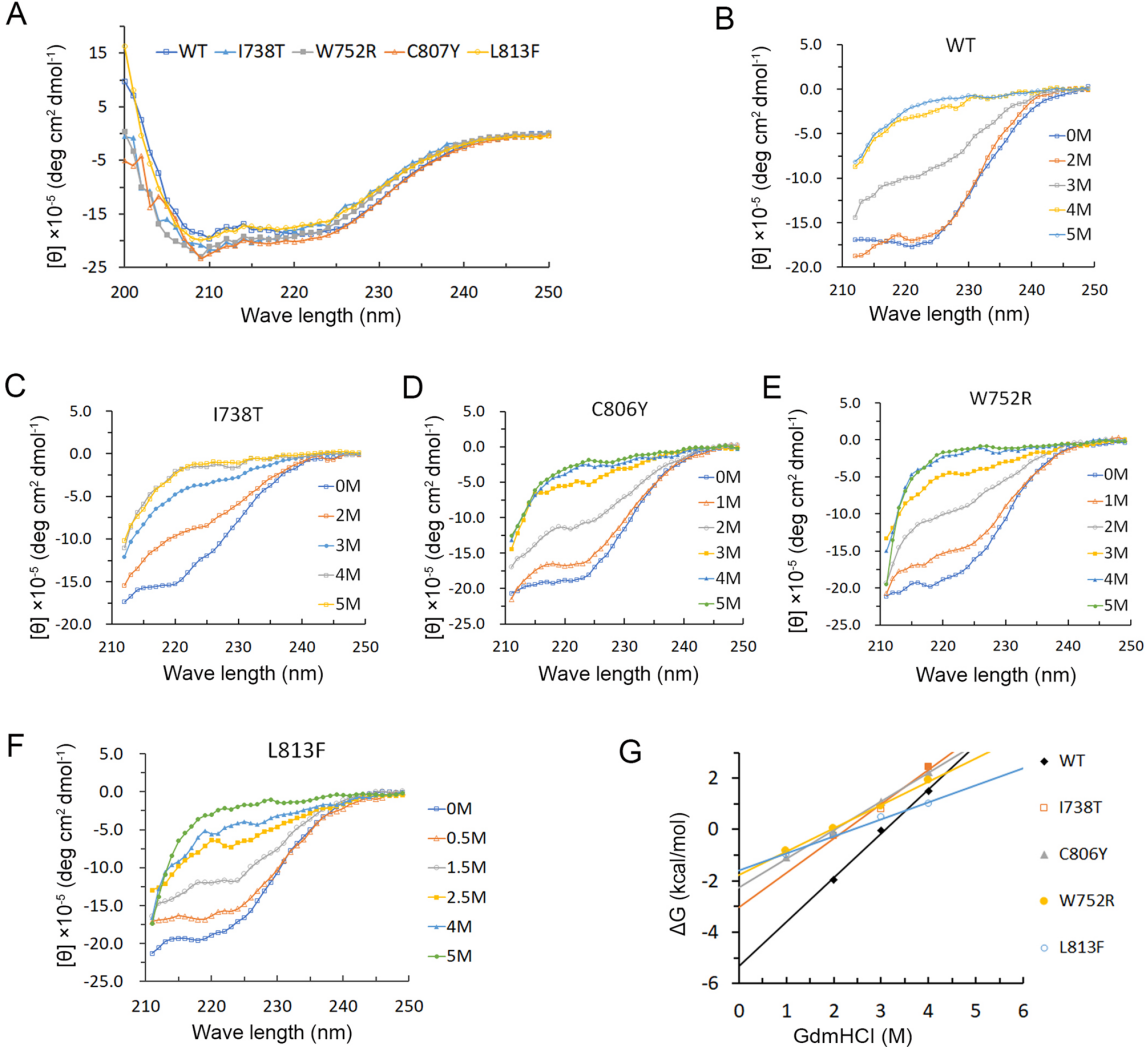
GdmHCl induced AR-LBD WT and mutant protein denaturation. (**A**) Circular dichroism spectra of AR-LBD WT and mutants at 25 ℃. (**B**–**F**) Changes of ellipticities of AR-LBD WT and mutants with increasing concentration of GdmHCl at 25 ℃. (**G**) The fitted folding free energies of AR-LBD WT and mutants using the two-state model by the linear extrapolation method.

In the room termperature, the AR-LBD WT and its mutants have similar CD spectra (200–250 nm, Fig. 8A), possessing the characteristic of α-helical proteins. We first tried to measure the temperature denaturation curves of AR-LBD WT and its mutants. However, since some mutants were observed to aggregate after elevating temperature, the measurement of the temperature-dependent folding stability by CD spectra looks unreliable. Therefore, we measured the stability of AR-LBD and its mutants by chemical denaturation.

Figure 8B–F shows the change of CD spectrum of AR-LBD WT and its mutant proteins with different concentrations of GdmHCl. Because GdmHCl itself has absorption below 210 nm, so we used the change of CD ellipticity at 222 nm to measure the fraction of folded protein and calculate the folding free energy of AR-LBD variants.

AR-LBD WT is the most stable sample in this test. The transition midpoint (C_1/2_) of AR-LBD WT is at 3.0 M GdmHCl, which is higher than those C_1/2_ of disease mutants (1.9–2.2 M GdmHCl, Table 1).

**Table 1:** Measured folding free energy of AR_LBD WT and mutants.

AR-LBD	Disease	m value	C_1/2[GdmHCl]_	ΔG_0_	∆∆G (FoldX)	∆∆G (experiments)	Solubility
WT	Health	1.7 ± 0.1	3.0	− 5.3 ± 0.1	0	0	+++++
I738T	PAIS	1.3 ± 0.1	2.2	− 3.0 ± 0.2	1.4	2.3	+++
C807Y	PAIS	1.1 ± 0.2	2.0	− 2.2 ± 0.1	6.2	3.1	+
W752R	CAIS	0.9 ± 0.1	1.9	− 1.7 ± 0.3	3.6	3.6	+
L813F	CAIS	0.6 ± 0.3	2.2	− 1.5 ± 0.1	6.3	3.8	+

Measured C_1/2[GdmHCl]_, folding free energy (ΔG_0_), standard error of folding free energy, change of folding free energy (∆∆G), m value, and standard error of m value of AR_LBD WT and mutants.

We further calculated the folding free energies of AR-LBD variants based on the GdmHCl denaturation experiment. The linear relationship between ΔG and GdmHCl concentration is shown in Fig. 8G. The intercept (ΔG_0_) at zero GdmHCl concentration gives the extrapolated folding free energy at physiological condition. The slope m represents the sensitivity of protein stability to denaturant. These values are given in Table 1.

The fitted folding free energy ΔG_0_ of AR-LBD WT is − 5.3 kcal/mol, while those of I738T, C807Y, W752R and L813F mutants are higher (less stable) by 2.3–3.8 kcal/mol (ΔΔ*G*). W752R and L813F, mutations of CAIS, compared to PAIS mutations I738T and C807Y, have greater changes in folding free energy. We proposed that AR mutants with less stabilities can lead to more severe androgen insensitivity syndrome. In summary, the experimental results are consistent with the FoldX prediction results.

The m value can be interpreted by the free energy change of the folded and unfolded protein being transferred from water to 1 M GdmHCl^61^. The m values of I738T, C807Y, W752R and L813F mutants were 1.3, 1.1, 0.9 and 0.6, respectively, which were lower than the m value of AR-LBD WT (1.7). The decrease of the m value indicates two possibilities: (1) the mutation increases the solvent accessibility of the hydrophobic residue in the folded state. (2) The deletion of some hydrogen bonds in the mutant will weaken the hydrophobic interaction of certain residues, thereby increasing its accessibility or sensitivity to GdmHCl. The decreasing trend of m value of mutants is also related to the ΔΔG of these mutant proteins. The L813F mutant with the smallest m value possessed the greatest decrease in protein stability (ΔΔG = 3.8 kcal/mol). The I738T with the smallest m value reduction has the smallest stability reduction (ΔΔG = 2.3 kcal/mol).

## Conclusion and outlook

In summary, for the first time through computer-based analysis, we found that mutations associated with human inherited diseases tend to have a significantly greater impact on protein stability than polymorphisms. Using computer-based analysis and GdmHCl denaturation experiments, we found that changes in protein folding stability are correlated with patient phenotypes. The change of folding free energy of the AR-LBD mutants predicted by FoldX are consistent with the measured folding free energy changes by GdmHCl denaturation experiments, which further supports the reliability of our conclusion. Therefore, this paper clearly demonstrates the importance of AR protein stability in the pathogenicity of hereditary diseases (AIS), and provides reference for clinic diagnosis.

In addition to predicting pathogenicity and improving diagnosis, this AR protein stability studies provide new opportunities for the treatment of AIS. Many pathogenic variations might be adequately functional, but are degraded by protein quality control system due to mild instability^62^. For these pathogenic variations, it may be possible to rescue protein function by preventing their recognition or degradation by the protein quality control system^62^. If the degradation of these protein variants is inhibited, it is possible to avoid pathogenicity. In addition, some pathogenic variations may be so unstable that even inhibition of their degradation is not sufficient to rescue their stability and function. These variants can restore protein function by stabilizing the protein with small molecules that bind directly to the unstable protein variants^63^. Small molecules compounds have been shown to rescue function of pathogenic variations, such as in mutants CFTR^64^ and TP53^65^.

## Methods

### Data set

We extracted AR variations from the following four databases: ARDB (The androgen receptor gene mutations database: 2012 update; https://androgendb.mcgill.ca), HGMD (Human Gene Mutation Database, 2015), Cosmic (2018.10.01), 1,000 genome;

*FoldX* A structure-based method for the prediction of free energy changes upon protein variations. Here we used FoldX that exploits both sequence and structural information to predict the protein stability changes upon single point mutation. When predicting the ΔΔG associated with a variation, positive value indicates that the protein is destabilized, and a negative value indicates that the protein is stable.

### Construction of AR mutants, protein overexpression and purification

The sequence of human AR cDNA (NCBI accessiong number: CCDS14387.1) encoding 664-920aa was cloned into pET28a expression vector. Site-directed mutagenesis was prepared using the procedure provided by the QuickChange site-directed mutagenesis kit. All constructs were verified by DNA sequencing. In the expression of WT AR and mutants, when the OD_600nm_ of the culture reached 0.5, DHT (dihydrotestosterone) was added with the final concentration of 30 μM. After 15 min, 0.1 mM IPTG was added to induce protein expression at 16 °C overnight. The cells were collected by centrifugation at 5,000g for 10 min at 4 °C. The centrifuged cells were then resuspended and sonicated in 20 mM Tris buffer containing 500 mM NaCl and 50 mM imidazole (pH8.0). Debris was removed by centrifugation at 20,000g for 30 min. The supernatant was loaded into a Ni–NTA column and the desired fraction was eluted with 300 mM imidazole. The eluent was loaded into a Superdex 75 column equilibrated with a buffer (20 mM Tris–HCl, pH 7.5, 200 mM NaCl, 1 mM EDTA, 10 mM 2-ME). The collected fractions were concentrated to about 0.5–2 mg/ml for CD measurement. The protein concentration was determined by using the calculated extinction coefficient at 280 nm.

### Circular dichroism (CD) measurements

CD measurement is performed on a Chirascan spectrometer. All CD measurements were performed in buffer (20 mM Tris–HCl, pH 7.5, 200 mM NaCl, 1 mM EDTA, 10 mM 2-ME). For the GdmHCl induced denaturation experiments, 70 μg/ml of AR-LBD WT and mutant proteins with different GdmHCl concentrations were prepared and equilibrated at 25 °C for 1 h. The CD signal was measured at a path of 0.1 cm, and three independent measurement results were averaged.

### Two-state analysis of GdmHCl denaturation

A two-state hypothesis was used to fit the denaturation curve of GdmHCl. The folding free energy of AR-LBD WT and mutant proteins without GdmHCl is estimated by a linear extrapolation:

$$\alpha_{i} = \frac{{\left[ {\theta_{i} \left] { {-} } \right[\theta_{{\text{U}}} } \right]}}{{\left[ {\theta_{{\text{F}}} \left] { - } \right[\theta_{{\text{U}}} } \right]}}$$

where [*θ*_*i*_] is the ellipticity at the *i*th gdmHCl concentration, [*θ*_F_] is the ellipticity of the protein completely folded, [*θ*_U_] is the ellipticity of the protein in 5 M GdmHCl. It is assumed that the protein in 5 M GdmHCl has been fully unfolded.

$$K_{i} = \frac{{{\upalpha }_{i} }}{{1 - {\upalpha }_{i} }}$$

where K_i_ is the folding constant of the monomer protein at the *i*th GdmHCl concentration, which can be calculated by the folding fraction α_*i*_. The free energy of protein folding can be estimated by the following equation:

$$\Delta G_{{\text{F}}} = - RT{\ln}K_{{\text{F}}}$$

where R is the gas constant, T is the absolute temperature, and *K*_F_ is the folding constant of monomer protein, which can be calculated by the function *K*_F_ = [F]/[U], where [F] and [U] represent respectively folded and unfolded fractions.

$$\Delta Gi = \Delta G_{0} + m\left[ {{\text{GdmHCl}}} \right]$$

The free energy of protein folding is a linear function of GdmHCl concentration, where Δ*G*_*i*_ is the free energy of protein at the *i*th GdmHCl concentration, and Δ*G*_0_ is the free energy of protein folding without GdmHCl.

## References

[1] Yue P, Li Z, Moult J (2005). Loss of protein structure stability as a major causative factor in monogenic disease. J. Mol. Biol..

[2] Karr JR (2012). A whole-cell computational model predicts phenotype from genotype. Cell.

[3] Socha RD, Tokuriki N (2013). Modulating protein stability—Directed evolution strategies for improved protein function. FEBS J..

[4] Goldstein RA (2008). The structure of protein evolution and the evolution of protein structure. Curr. Opin. Struct. Biol..

[5] Magliery TJ (2015). Protein stability: Computation, sequence statistics, and new experimental methods. Curr. Opin. Struct. Biol..

[6] Teilum K, Olsen JG, Kragelund BB (1814). Protein stability, flexibility and function. Biochem. Biophys. Acta..

[7] Mainwaring WI (1977). The mechanism of action of androgens. Monogr. Endocrinol..

[8] Davey RA, Grossmann M (2016). Androgen receptor structure, function and biology: From bench to bedside. Clin. Biochem. Rev..

[9] Shukla GC, Plaga AR, Shankar E, Gupta S (2016). Androgen receptor-related diseases: What do we know?. Andrology.

[10] Galani A, Kitsiou-Tzeli S, Sofokleous C, Kanavakis E, Kalpini-Mavrou A (2008). Androgen insensitivity syndrome: Clinical features and molecular defects. Hormones.

[11] Hughes IA (2012). Androgen insensitivity syndrome. Lancet.

[12] Gottlieb B, Beitel LK, Nadarajah A, Paliouras M, Trifiro M (2012). The androgen receptor gene mutations database: 2012 update. Hum. Mutat..

[13] Finsterer J (2009). Bulbar and spinal muscular atrophy (Kennedy's disease): A review. Eur. J. Neurol..

[14] Izumi K, Mizokami A, Lin WJ, Lai KP, Chang C (2013). Androgen receptor roles in the development of benign prostate hyperplasia. Am. J. Pathol..

[15] Bousema JT (2000). Polymorphisms in the vitamin D receptor gene and the androgen receptor gene and the risk of benign prostatic hyperplasia. Eur. Urol..

[16] Yeh S (2003). Abnormal mammary gland development and growth retardation in female mice and MCF7 breast cancer cells lacking androgen receptor. J. Exp. Med..

[17] Peters KM (2012). Androgen receptor expression predicts breast cancer survival: The role of genetic and epigenetic events. BMC Cancer.

[18] Kalra M, Mayes J, Assefa S, Kaul AK, Kaul R (2008). Role of sex steroid receptors in pathobiology of hepatocellular carcinoma. World J. Gastroenterol..

[19] Rogers AB (2007). Hepatocellular carcinoma associated with liver-gender disruption in male mice. Can. Res..

[20] Ma WL, Lai HC, Yeh S, Cai X, Chang C (2014). Androgen receptor roles in hepatocellular carcinoma, fatty liver, cirrhosis and hepatitis. Endocr. Relat. Cancer.

[21] Tan MH, Li J, Xu HE, Melcher K, Yong EL (2015). Androgen receptor: Structure, role in prostate cancer and drug discovery. Acta Pharmacol. Sin..

[22] Cato AC, Henderson D, Ponta H (1987). The hormone response element of the mouse mammary tumour virus DNA mediates the progestin and androgen induction of transcription in the proviral long terminal repeat region. EMBO J..

[23] Ham J, Thomson A, Needham M, Webb P, Parker M (1988). Characterization of response elements for androgens, glucocorticoids and progestins in mouse mammary tumour virus. Nucleic Acids Res..

[24] Pandini G (2005). Androgens up-regulate the insulin-like growth factor-I receptor in prostate cancer cells. Cancer Res..

[25] Kim J, Coetzee GA (2004). Prostate specific antigen gene regulation by androgen receptor. J. Cell. Biochem..

[26] Wang LG, Liu XM, Kreis W, Budman DR (1997). Down-regulation of prostate-specific antigen expression by finasteride through inhibition of complex formation between androgen receptor and steroid receptor-binding consensus in the promoter of the PSA gene in LNCaP cells. Cancer Res..

[27] Bastus NC (2010). Androgen-induced TMPRSS2:ERG fusion in nonmalignant prostate epithelial cells. Cancer Res..

[28] Cai C, Wang H, Xu Y, Chen S, Balk SP (2009). Reactivation of androgen receptor-regulated TMPRSS2:ERG gene expression in castration-resistant prostate cancer. Cancer Res..

[29] Yu J (2010). An integrated network of androgen receptor, polycomb, and TMPRSS2-ERG gene fusions in prostate cancer progression. Cancer Cell.

[30] Schymkowitz J (2005). The FoldX web server: An online force field. Nucleic Acids Res..

[31] Buss O, Rudat J, Ochsenreither K (2018). FoldX as protein engineering tool: Better than random based approaches?. Computat. Struct. Biotechnol. J..

[32] Capriotti E, Fariselli P, Rossi I, Casadio R (2008). A three-state prediction of single point mutations on protein stability changes. BMC Bioinform..

[33] Dehouck Y, Kwasigroch JM, Gilis D, Rooman M (2011). PoPMuSiC 2.1: A web server for the estimation of protein stability changes upon mutation and sequence optimality. BMC Bioinform..

[34] Pires DE, Ascher DB, Blundell TL (2014). mCSM: Predicting the effects of mutations in proteins using graph-based signatures. Bioinformatics.

[35] Laimer J, Hiebl-Flach J, Lengauer D, Lackner P (2016). MAESTROweb: A web server for structure-based protein stability prediction. Bioinformatics.

[36] Guerois R, Nielsen JE, Serrano L (2002). Predicting changes in the stability of proteins and protein complexes: A study of more than 1000 mutations. J. Mol. Biol..

[37] Zhou H, Zhou Y (2002). Distance-scaled, finite ideal-gas reference state improves structure-derived potentials of mean force for structure selection and stability prediction. Protein Sci. Publ. Protein Soc..

[38] Capriotti E, Fariselli P, Casadio R (2005). I-Mutant2.0: Predicting stability changes upon mutation from the protein sequence or structure. Nucleic Acids Res..

[39] Parthiban V, Gromiha MM, Schomburg D (2006). CUPSAT: Prediction of protein stability upon point mutations. Nucleic Acids Res..

[40] Yin S, Ding F, Dokholyan NV (2007). Eris: An automated estimator of protein stability. Nat. Methods.

[41] Quan L, Lv Q, Zhang Y (2016). STRUM: Structure-based prediction of protein stability changes upon single-point mutation. Bioinformatics.

[42] Zhang Z (2012). Predicting folding free energy changes upon single point mutations. Bioinformatics.

[43] Sasaki M (2003). The polyglycine and polyglutamine repeats in the androgen receptor gene in Japanese and Caucasian populations. Biochem. Biophys. Res. Commun..

[44] Hsing AW (2000). Polymorphic CAG and GGN repeat lengths in the androgen receptor gene and prostate cancer risk: A population-based case–control study in China. Cancer Res..

[45] Choong CS, Kemppainen JA, Zhou ZX, Wilson EM (1996). Reduced androgen receptor gene expression with first exon CAG repeat expansion. Mol. Endocrinol..

[46] Lavery DN, McEwan IJ (2008). Structural characterization of the native NH2-terminal transactivation domain of the human androgen receptor: A collapsed disordered conformation underlies structural plasticity and protein-induced folding. Biochemistry.

[47] Reid J, Kelly SM, Watt K, Price NC, McEwan IJ (2002). Conformational analysis of the androgen receptor amino-terminal domain involved in transactivation. Influence of structure-stabilizing solutes and protein–protein interactions. J. Biol. Chem..

[48] McEwan IJ, Gustafsson J (1997). Interaction of the human androgen receptor transactivation function with the general transcription factor TFIIF. Proc. Natl. Acad. Sci. USA.

[49] Bevan CL, Hoare S, Claessens F, Heery DM, Parker MG (1999). The AF1 and AF2 domains of the androgen receptor interact with distinct regions of SRC1. Mol. Cell. Biol..

[50] Schaufele F (2005). The structural basis of androgen receptor activation: Intramolecular and intermolecular amino–carboxy interactions. Proc. Natl. Acad. Sci. USA.

[51] He B (2004). Structural basis for androgen receptor interdomain and coactivator interactions suggests a transition in nuclear receptor activation function dominance. Mol. Cell.

[52] Shaffer PL, Jivan A, Dollins DE, Claessens F, Gewirth DT (2004). Structural basis of androgen receptor binding to selective androgen response elements. Proc. Natl. Acad. Sci. USA.

[53] Ni L (2013). Androgen induces a switch from cytoplasmic retention to nuclear import of the androgen receptor. Mol. Cell. Biol..

[54] Matias PM (2000). Structural evidence for ligand specificity in the binding domain of the human androgen receptor. Implications for pathogenic gene mutations. J. Biol. Chem..

[55] Rawla P (2019). Epidemiology of prostate cancer. World J. Oncol..

[56] Beitel LK, Scanlon T, Gottlieb B, Trifiro MA (2005). Progress in Spinobulbar muscular atrophy research: Insights into neuronal dysfunction caused by the polyglutamine-expanded androgen receptor. Neurotox. Res..

[57] Lek M (2016). Analysis of protein-coding genetic variation in 60,706 humans. Nature.

[58] Gray VE, Hause RJ, Fowler DM (2017). Analysis of large-scale mutagenesis data to assess the impact of single amino acid substitutions. Genetics.

[59] Hartl FU, Bracher A, Hayer-Hartl M (2011). Molecular chaperones in protein folding and proteostasis. Nature.

[60] Nielsen SV (2017). Predicting the impact of Lynch syndrome-causing missense mutations from structural calculations. PLoS Genet..

[61] Auton M, Bolen DW (2005). Predicting the energetics of osmolyte-induced protein folding/unfolding. Proc. Natl. Acad. Sci. USA.

[62] Kampmeyer C (2017). Blocking protein quality control to counter hereditary cancers. Genes Chromosom. Cancer.

[63] Pereira DM, Valentao P, Andrade PB (2018). Tuning protein folding in lysosomal storage diseases: The chemistry behind pharmacological chaperones. Chem. Sci..

[64] Van Goor F (2011). Correction of the F508del-CFTR protein processing defect in vitro by the investigational drug VX-809. Proc. Natl. Acad. Sci. USA.

[65] Joerger AC, Fersht AR (2016). The p53 pathway: Origins, inactivation in cancer, and emerging therapeutic approaches. Annu. Rev. Biochem..

